# Molecular characterization of Leber congenital amaurosis in Koreans

**Published:** 2008-08-04

**Authors:** Moon-Woo Seong, Seong Yeon Kim, Young Suk Yu, Jeong-Min Hwang, Ji Yeon Kim, Sung Sup Park

**Affiliations:** 1 Department of Laboratory Medicine, Seoul National University Hospital and Clinical Research Institute, Seoul, Korea;; 2 Department of Laboratory Medicine, National Cancer Center, Goyang, Korea; 3 Department of Ophthalmology, Seoul National University Hospital, Seoul, Korea; 4 Department of Ophthalmology, Seoul National University Bundang Hospital, Seongnam, Korea

## Abstract

**Purpose:**

Leber congenital amaurosis (LCA) is the most severe form of inherited retinal dystrophy, and invariably leads to blindness. LCA is a genetically and clinically heterogenous disorder. Although more than nine genes have been found to be associated with LCA, they only account for about half of LCA cases. We performed a comprehensive mutational analysis on nine known genes in 20 unrelated patients to investigate the genetic cause of LCA in Koreans.

**Methods:**

All exons and flanking regions of the nine genes (*AIPL1, CRB1, CRX, GUCY2D, RDH12, RPE65, RPGRIP1, LRAT,* and *TULP1*) were analyzed by direct sequencing. We also screened our patients for the common *CEP290*: c.2991+1655A>G mutation found in Caucasian.

**Results:**

Six different mutations including four novel ones were identified in three patients (15.0%): one frameshift, one nonsense, one splicing, and three missense mutations. These patients were compound heterozygotes and harbored two different mutations in *CRB1*, *RPE65,* and *RPGRIP1*, respectively. We identified three novel unclassified missense variants in *RPGRIP1 *of the three patients. These patients were heterozygous for each variant and did not have a large deletion or duplication in the same gene.

**Conclusions:**

This comprehensive mutational analysis shows marked genetic heterogeneity in Korean LCA patients and reveals a mutation spectrum that differs from those previously reported. In turn, this suggests that a different strategy should be used for the molecular diagnosis of LCA in Koreans.

## Introduction

Leber congenital amaurosis (LCA; OMIM 204000), is the most severe form of all inherited retinal dystrophies, and is an important cause of congenital blindness in many countries [1,2]. Its incidence has been estimated at 2–3 per 100,000 live births, and it is known that LCA accounts for 5% of all inherited retinal dystrophies, and for up to 20% of children attending schools for the blind worldwide.

LCA is a clinically and genetically heterogenous disorder. Early onset blindness during the first year of life (especially before six months), ocular features like oculodigital signs (eye poking, rubbing, and pressing), sluggish pupillary reaction, and extinguished or severely reduced ERG are accepted highly suggestive criteria, but none of these are diagnostic for LCA [1]. In addition to ocular symptoms, systemic symptoms such as neurodevelopmental delay can be associated with LCA. However, some systemic diseases, such as Senior-Loken syndrome, Conorenal syndrome, and Joubert syndrome, can manifest ocular symptoms, which complicate the differential diagnosis [3,4]. Alternatively, early-onset retinal dystrophies like retinitis pigmentosa (RP; OMIM 268000) and cone-rod dystrophy (CRD; OMIM 600624) may have clinical features resembling those of LCA.

Nine genes, i.e., *GUCY2D* (LCA1), *RPE65* (LCA2), *AIPL1* (LCA4), *RPGRIP1, LCA5* (LCA6), *CRB1* (LCA7), *CRX* (LCA8), *RDH12,* and *CEP290* (LCA10) are generally accepted to be implicated in LCA, and three additional genes (*TULP1, LRAT*, and *IMPDH1*) and two loci (LCA3 and LCA9) may also be associated with the disease (RetNet, Genetests). However, LCA may be associated with many more genes: only an estimated 50% of cases have been diagnosed by molecular methods even in large studies, and about 130 genes are known to be implicated in inherited retinal diseases [5]. Some genes related with LCA are involved in other inherited retinal diseases, such as RP and CRD, and thus these diseases may be viewed as a spectrum of genetically related diseases [6,7].

The clinical and genetic heterogeneity of LCA hampers its routine molecular diagnosis. The establishment of phenotype-genotype correlations and the development of a high-throughput screening method would offer a means of overcoming these difficulties. The comprehensive mutational analysis is required to both establish genotype-phenotype correlations and determine mutation distribution patterns, but few such studies have been conducted to date [5,8]. Moreover, those results mainly came from Caucasian, so comprehensive mutational analysis in non-Caucasian can be helpful to understand pathogenic mechanism of LCA. Here, we report the results of a comprehensive mutational analysis conducted on nine known LCA genes in 20 Korean LCA patients.

## Methods

### Subjects

A total of 20 unrelated patients were recruited from the ophthalmology clinics at Seoul National University Hospital and Seoul National University Bundang Hospital from 1999 to 2007. The median age of patients at initial diagnosis was 8 months (range 3 to 33) and male to female ratio was 2:3. Informed consent was obtained from all patients or their legal guardians for the provision of clinical information and blood samples. All patients received a detailed ophthalmic examination including electroretinogram and was diagnosed with LCA based on the following criteria, suggested by De Laey [9]: early onset blindness or severe visual impairment during the first year of life (especially before six months), with oculodigital signs (eye poking, rubbing, and pressing); an extinguished or severely reduced ERG; and the exclusion of other systemic diseases.

The mutational analysis included 170 healthy individuals as a control for a 1% polymorphism [10].

### Mutational analysis

#### Sequence analysis of nine genes

Genomic DNA was immediately extracted from peripheral blood using Gentra PureGene DNA isolation kits (Gentra Systems, Inc. Minneapolis, MN). The full sequence of nine genes that have been associated with LCA or an LCA-like phenotype were analyzed, i.e., seven genes associated with LCA: *AIPL1, CRB1, CRX, GUCY2D, RDH12, RPE65,* and *RPGRIP1,* and two genes associated with an LCA-like phenotype: *LRAT,* and *TULP*. PCR was performed on patient genomic DNA using primers designed to flank the splice junctions of coding exons. The PCR parameters were as follows: 95 °C for 5 min, followed by 35 cycles of 95 °C for 30 s, 60 °C for 30 s, and 72 °C for 1 min. Amplified products were sequenced bidirectionally on an ABI Prism 3100 Genetic Analyzer (Applied Biosystems, Foster City, CA), then analyzed using Sequencher software (Gene Codes Co, Ann Arbor, MI).

#### c.2991+1655A>G mutation of CEP290

In addition to full sequencing of nine genes, we performed allele-specific PCR. This was to determine whether c.2991+1655A>G, an intronic mutation in *CEP290* and described as one of the most frequent causes of LCA in a Caucasian, could also be a common cause in the Korean population [11].

#### Gene dosage analysis

In the case of a single heterozygote with one mutation, we performed semiquantitative PCR to exclude the possibility of a large deletion or duplication in the gene concerned. Each exon of *RPGRIP1* and the reference gene, *B2MG*, were co-amplified with fluorescence-labeled primers through 18 limited cycles. Then labeled PCR products were analyzed on the ABI Prism 3100 Genetic Analyzer, and the heights of the peaks of interest were measured with the ABI Prism Data Collection Software (v2.0). Normalized gene dosage for each exon was determined by using the following equation:

Gene dosage=[Peak_target_(patient)/Peak_reference_(patient)]/[Peak_target_(control)/Peak_reference_(control)]

### Determination of significance of novel sequence variations

#### Allele frequency in control subjects

To investigate allele frequencies, we screened control subjects by denaturing high-pressure liquid chromatography (dHPLC). DNA, pooled from three control subjects, was amplified. Next, PCR products were denatured for 10 min at 95 °C and then gradually reannealed by decreasing temperatures from 95 °C to 25 °C over 30 min. PCR products were eluted at a flow rate of 0.9 ml/min on the Wave 3500 (Transgenomics, Omaha, NE). Pooled DNA samples displaying an abnormal profile were analyzed by direct sequencing to determine the specific genotype of each subject.

#### Information from amino acids and proteins

Generally, in genetic mutation studies such as the present study, it is critical to determine whether novel missense variations are likely to be harmful to protein function or structure. However, functional analysis is not always available to investigate the effect of a missense variation on a protein. We have predicted the functional effect of a novel missense variation using information from the characteristics of the amino acids substituted, interspecies amino acid conservation using ClustalW [12], and protein structural information from Uniprot.

#### In-silico prediction of novel missense variation using different software

We compared the aforedescribed results with those obtained using three protein function prediction software: Polyphen [13], SIFT [14], and PMut [15]. All three prediction software packages have been previously applied to various disease-gene models [16-18].

## Results

### Mutations

We identified six different mutations in three patients (15%), in *CRB1* (5%), *RPE65* (5%), and *RPGRIP1* (5%; Table 1). No homozygous mutations were found in this study. All three patients had a compound heterozygous mutation: c.271C>T (R91W) and c.858+1G>T (IVS8+1G>T) in *RPE65* (case 5); c.1892A>T (H631P) and c.3560_3566delAAGGCCG in *RPGRIP1* (case 13); and c.998G>A (G333D) and c.1576C>T (R526X) in *CRB1* (case 17).

**Table 1:** Characterization of mutations and novel unclassified variants identified in this study

** ** **Case #**	** ** **Gene**	** ** **Mutation**	**Characterization of variant**	**Frequency in control**	**Amino acid Conservation**	** ** **Domain**	**In-silico analysis**		
							**Polyphen**	**SIFT**	**PMut**
5	*RPE65*	c.271C>T (R91W)*	Missense/Pathogenic						
		c.858+1G>T (IVS8+1G>T)*	Splicing/Pathogenic						
11	*RPGRIP1*	c.1295C>T (S432F)	Missense/Unclassified	0.003	Some species	Coiled coil region	Damaging	Not tolerated	Pathological
6	*RPGRIP1*	c.1802C>G (S601W)	Missense/Unclassified	<0.01	Some species	C2 domain	Damaging	Not tolerated	Pathological
13	*RPGRIP1*	c.1892A>T (H631P)	Missense/Pathogenic	<0.01	Well conserved	C2 domain	Damaging	Tolerated	Pathological
		c.3560_3566delAAGGCCG	Frameshift/Pathogenic						
12	*RPGRIP1*	c.3170A>T (H1057L)	Missense/Unclassified	0.006	Some species	RPGR interacting domain	Damaging	Tolerated	Pathological
17	*CRB1*	c.998G>A (G333D)	Missense/Pathogenic	<0.01	Well conserved	EGF-like domain	Damaging	Not tolerated	Neutral
		c.1576C>T (R526X)	Nonsense/Pathogenic						

Mutations and novel unclassified variants are presented. The asterisk indicates that this has been previously reported elsewhere as a pathologic mutation. The sequence variations without the asterisk are novel ones with further analyses supporting or excluding pathogenicity including frequency in normal control, amino acid conservation and in-silico prediction using softwares. We classified H631P in *RPGRIP1* and G333D in *CRB1* among five missense variants as pathogenic mutations, but other missense ones as unclassified variants.

All six mutations uniquely occurred in families. Two mutations in *RPGRIP1* and two in *CRB1* were novel, whereas two mutations found in *RPE65* have been reported previously [19,20]. Two of four novel mutations produced null alleles: c.3560_3566delAAGGCCG (premature protein translation termination at codon 1195) and c.1576C>T (R526X). Segregation of disease alleles was confirmed in case 13, for whom DNA samples from both parents were available. We classified the other two novel missense variations as pathogenic mutations because each was accompanied by a null allele and was predicted to be harmful to protein structure or function on prediction analysis.

Case 13, who had novel mutations in *RPGRIP1*, had a history of photophobia and displayed peripheral hyperpigmentation in the retina. The posterior pole and disc had a relatively normal appearance. Visual acuity was 20/500 OD and 20/500 OS. Case 17, who had novel mutations in *CRB1*, had a history of night blindness and diffuse hyperpigmentation in the retina, with vascular attenuation. Visual acuity was 20/300 OD and hand motion OS. These findings were similar to the genotype-phenotype correlations suggested by Hanein et al. [5].

**Figure 1:**
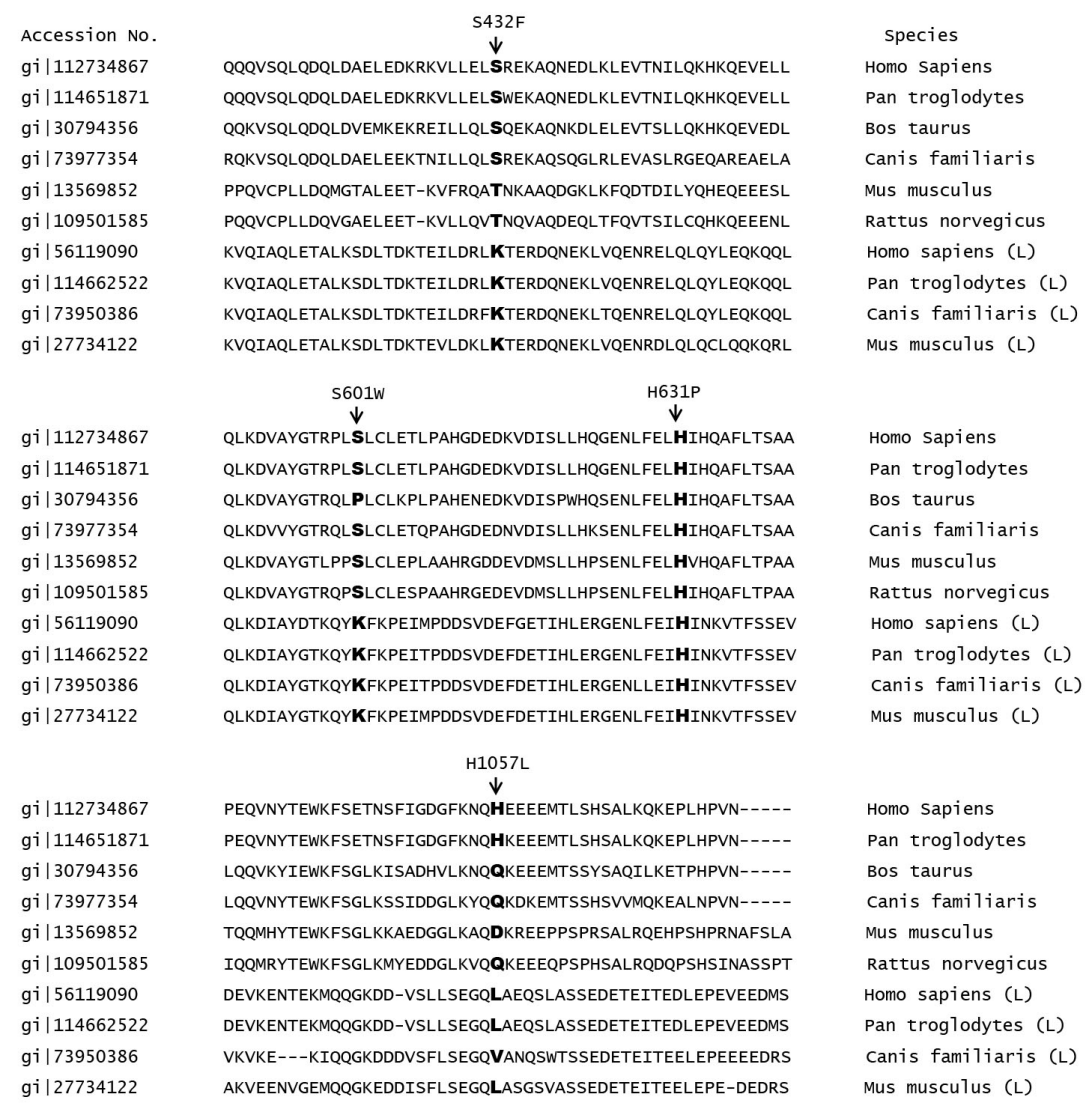
Multiple alignments using ClustalW and amino acid conservation of four novel missense sequence variations identified in *RPGRIP1*: c.1295C>T (S432F), c.1802C>G (S601W), c.1892A>T (H631P), and c.3170A>T (H1057L). The first six amino acid sequences in each segment represent RPGRIP1 proteins of several species, and the last four denoted with (L) represent RPGRIP1-like proteins. Alignment results show that histidine at codon 631 is highly conserved, but amino acids at codon 432, 601, and 1057 are poorly conserved.

The two novel missense variations were not found among 170 control subjects, which showed allele frequencies of <0.01 for all variations (Table 1). We analyzed amino acid conservation for the genes concerned in *Homo sapiens, Pan troglodytes, Bos taurus*, *Canis familiaris*, *Mus musculus*, and *Rattus norvegicus*. Two missense variations were well conserved across these species and homologous proteins (Figure 1). H631P was located in the structurally important C2 domain [21] and G333D was located in an epidermal growth factor (EGF)-like domain, near a disulfide bond between codon 327 and 336. Moreover, all of the aforedescribed substituted amino acids were quite different from the original amino acids in terms of their physicochemical characteristics. The BLOSUM62 [22] matrix score was also negative for two missense variations, which supports their pathogenic potential, and Polyphen, SIFT, and PMut produced similar results. These variations were predicted to be pathogenic by two or more of these prediction tools. Therefore, we considered c.1892A>T (H631P) in *RPGRIP1*, and c.998G>A (G333D) in *CRB1* as pathogenic mutations (Table 1).

### Unclassified missense variants

Interestingly, we identified three novel missense variations only in *RPGRIP1*: c.1295C>T (S432F), c.1802C>G (S601W), and c.3170A>T (H1057L). All patients with these missense variants were heterozygous for each variant and a second mutation, and the presence of a large deletion or duplication in the same gene were excluded in these patients.

Three variants were located in structurally important regions: S432F in the coiled-coil region, S601W in the C2 domain, and H1057L in the RPGR interacting domain. All substitutions represented negative BLOSUM62 [22] matrix score and were predicted to be pathogenic using the prediction software packages. c.1295C>T (S432F) and c.3170A>T (H1057L) were also found in control subjects (Table 1). Amino acid conservation at these positions was restricted to some species (Figure 1), suggesting a possibility of rare polymorphism. c.1802C>G (S601W) was not found among 170 control subjects, although serine at codon 601 was not well conserved among different species. Therefore, it is uncertain at this point whether c.1802C>G (S601W) is a rare polymorphism or not.

### Polymorphisms

In addition to the aforedescribed mutations and unclassified variants, we observed 82 sequence variations, of which 24 were located in exons and 58 in introns (Table 2). The following three among 13 nonsynonymous sequence variations were novel: c.2809G>A (A937T) in *CRB1*, c.460A>G (T154A) in *CRX*, and c.783G>C (K261N) in *TULP1*. A nonsynonmous sequence variation in *CRB1*, c.2809G>A (A937T), was found in EGF-like domain 14, but it was felt that this substitution was unlikely to impair protein function because the two amino acids have similar physicochemical characteristics. All three software tools predicted that this substitution would not be pathological (Polyphen score of 0.428, SIFT score of 0.70, and PMut score of 0.22). A nonsynonymous sequence variation in *CRX,* c.460A>G (T154A) was also considered to be a polymorphic sequence variation, because it is located outside the homeobox domain (35–101), even though the amino acid is well conserved. The three programs concurred that substitution is unlikely to be pathologic (Polyphen score of 1.449, SIFT [score of 0.26, and PMut score of 0.25). We did not find these two nonsynonymous mutations in control subjects, and therefore, we consider them rare polymorphic sequence variations. Finally, c.783G>C (K261N) in *TULP1* was frequently found in controls and patients.

**Table 2:** Polymorphic sequence variations identified in this study.

**Gene**	**Variation**	**Allele frequency**	**Reference**	**Gene**	**Variation**	**Allele frequency**	**Reference**	**Gene**	**Variation**	**Allele frequency**	**Reference**
*GUCY2D*	c.154G>T (A52S)	0.75	[23]	RPGRIP1	c.287C>A (P96Q)	0.05	[26]	*CRX*	IVS3-67G>A	0.08	
	c.741C>T (H247H)	0.15			c.450C>G (L150L)	0.05			IVS3-131A>T	0.03	
	c.1371C>T (C457C)	0.15			c.574A>G (K192E)	0.3	[26]		c.460A>G (T154A)	0.03	
	IVS5+143C>T	0.03			IVS6-14_16delAAT	0.28		*RDH12*	IVS2+60G>A	0.4	
	IVS6+72C>T	0.03			IVS7+26T>C	0.65			IVS2-13insT	0.08	
	c.2101C>T (P701S)	0.18	[24]		IVS9-65G>A	0.35			IVS2-58A>G	0.03	
*RPE65*	c.-20G>A	0.03			IVS11-8C>T	0.03			IVS3-115G>C	1	
	IVS3-46G>A	0.15			IVS13+148delG	0.03			c.482G>A (R161Q)	0.08	[28]
	IVS9+112T>C	0.25			c.1797G>A (P599P)	0.03			c.570C>T (S190S)	0.03	
	c.1056G>A (E352E)	0.33			c.3097G>C (E1033Q)	0.43	[26]	*TULP1*	IVS1+57T>C	0.03	
	IVS12+20A>C	0.4			IVS21-148T>G	0.03			IVS1+58G>C	0.03	
*AIPL1*	c.1-106C>A	0.3			IVS21-27T>A	0.03			IVS1+62_67delAGTGGG	0.03	
	c.1-107G>A	0.03			IVS22+154A>G	0.1			IVS2+18G>A	0.08	
	IVS1+36C>T	0.08			IVS23+17delT	0.03			IVS2+154A>G	0.73	
	IVS1+45T>C	0.08		*CRB1*	IVS1-12A>T	0.83			IVS3+81G>C	0.53	
	IVS1+105C>A	0.08			c.747C>T (D249D)	0.03			c.200C>G (T67R)	0.85	[29]
	IVS1+148G>A	0.03			IVS3-64A>G	0.03			IVS4-29C>T	0.03	
	c.268G>C (D90H)	0.3	[25]		IVS3-35T>C	0.68			IVS5+26C>T	0.75	
	IVS2-14G>A	0.03			IVS4+35C>T	0.1			IVS5+170T>C	0.7	
	IVS2-10A>C	0.45			IVS4-53T>G	0.68			IVS7+108A>G	0.55	
	c.300A>G (L100L)	0.53			c.1410A>G (L470L)	1			IVS7-63G>A	0.8	
	IVS3-26T>C	0.1			c.2306G>A (R769H)	0.05	[27]		c.776T>C (I259T)	0.58	[30]
	IVS4+48G>A	0.5			IVS7-129C>A	0.03			c.783G>C (K261N)	0.83	
	IVS4-33C>T	0.33			c.2796G>A (P932P)	0.03			IVS8+83G>A	0.03	
	c.651A>G (P217P)	0.73			c.2809G>A (A937T)	0.03			IVS8-76T>C	0.13	
	IVS5+18G>A	0.08			IVS8+87C>G	0.03			IVS8-17G>C	0.28	
	IVS5+89C>T	0.33			IVS10+88insT	0.1			IVS13+90A>G	0.1	
									IVS13-87T>C	0.05	

All polymorphic sequence variations in nine genes are presented here. Allele frequency was estimated in the patient group. Ten among thirteen missense variants were previously reported as polymorphic variants elsewhere. Three novel ones including c.2809G>A in *CRB1*, c.460A>G in *CRX* and c.783G>C in *TULP1* were classified as polymorphic variants.

We identified 58 intronic sequence variations in patients. Intronic sequence variations flanking exon-intron boundaries potentially capable of affecting exon splicing were as follows: IVS2–14G>A (allele frequency, 0.03) and IVS5+18G>A (allele frequency, 0.08) in *AIPL1*, and IVS2–13insT (allele frequency, 0.08) in the *RDH12*, IVS2+18G>A (allele frequency, 0.08) in *TULP1*. However, we could not exclude the possibility of splice disruption because we had failed to recover the mRNA of concerned genes from peripheral blood cells.

## Discussion

The mutation spectrum revealed in this study shows marked genetic heterogeneity as well as different features from those found in previous studies. In previous studies except ones about *CEP290*, mutation in *GUCY2D* was most common (6%–21%), followed by *CRB1,* and *RPE65*, and the mutations in *RPGRIP1* accounted for less than 5% of all mutations [5,7,8]. In our series, however, neither *GUCY2D* mutation nor the intronic mutation, *CEP290*: c.2991+1665A>G was never found [11]. In addition, the molecular detection rate was only 15% in this study, despite the inclusion of all nine known genes, which is substantially lower than about 50% in other large studies. Finally, all three patients harboring two mutations were compound heterozygotes, and all mutations were restricted to families. This mutation spectrum suggests that there might be no founder mutation, but rather that Korean LCA patients show marked genetic heterogeneity. Our findings also mean that it will be difficult to develop an effective screening method, and that a search for new candidate genes is warranted.

We identified three novel unclassified variants in *RPGRIP1*. A possibility of pathogenic mutation remains questionable; patients heterozygous for each variant do not have a second mutation in the same gene, and functional effects of such a substitution is controversial on predictions. However, a large gene rearrangement or hidden mutation in the unscreened region could be complicated with these variants observed in this study. We excluded the possibilities of a large deletion or duplication using the gene dosage test, but we could not exclude the possibility of a hidden splice mutation because we had failed to recover the mRNA of *RPGRIP1* from peripheral blood cells. Mutation in another gene may have an additive effect to these variants of unknown significance. Interestingly, all these heterozygous missense variations were in *RPGRIP1*. Because RPGRIP1 protein closely interacts with RPGR in the retinal pigment epithelium and *RPGR* causes severe X-linked RP, a digenism by *RPGRIP1* and *RPGR* may be a potential cause of many heterozygotes in this study.

The locus heterogeneity and allelic heterogeneity of LCA necessitate the development of an effective screening tool, such as a microarray, or the establishment of genotype-phenotype correlations, and is also require comprehensive mutational analysis in this field. This study is not only one of a few reports of comprehensive mutational analysis but to our knowledge is also the most comprehensive one in the non-Caucasian. In summary, our study shows marked genetic heterogeneity in Korean LCA patients and reveals a mutation spectrum that differs from those previously reported, indicating a different strategy should be used for the molecular diagnosis of LCA in the Korean population.

## References

[1] Fazzi E, Signorini SG, Scelsa B, Bova SM, Lanzi G (2003). Leber's congenital amaurosis: an update.. Eur J Paediatr Neurol.

[2] Koenekoop RK (2004). An overview of Leber congenital amaurosis: a model to understand human retinal development.. Surv Ophthalmol.

[3] Fazzi E, Signorini SG, Uggetti C, Bianchi PE, Lanners J, Lanzi G (2005). Towards improved clinical characterization of Leber congenital amaurosis: neurological and systemic findings.. Am J Med Genet A.

[4] Casteels I, Spileers W, Demaerel P, Casaer P, De Cock P, Dralands L, Missotten L (1996). Leber congenital amaurosis–differential diagnosis, ophthalmological and neuroradiological report of 18 patients.. Neuropediatrics.

[5] Hanein S, Perrault I, Gerber S, Tanguy G, Barbet F, Ducroq D, Calvas P, Dollfus H, Hamel C, Lopponen T, Munier F, Santos L, Shalev S, Zafeiriou D, Dufier JL, Munnich A, Rozet JM, Kaplan J (2004). Leber congenital amaurosis: comprehensive survey of the genetic heterogeneity, refinement of the clinical definition, and genotype-phenotype correlations as a strategy for molecular diagnosis.. Hum Mutat.

[6] Allikmets R (2004). Leber congenital amaurosis: a genetic paradigm.. Ophthalmic Genet.

[7] Cremers FP, van den Hurk JA, den Hollander AI (2002). Molecular genetics of Leber congenital amaurosis.. Hum Mol Genet.

[8] Zernant J, Külm M, Dharmaraj S, den Hollander AI, Perrault I, Preising MN, Lorenz B, Kaplan J, Cremers FP, Maumenee I, Koenekoop RK, Allikmets R (2005). Genotyping microarray (disease chip) for Leber congenital amaurosis: detection of modifier alleles.. Invest Ophthalmol Vis Sci.

[9] De Laey JJ (1991). Leber's congenital amaurosis.. Bull Soc Belge Ophtalmol.

[10] Collins JS, Schwartz CE (2002). Detecting polymorphisms and mutations in candidate genes.. Am J Hum Genet.

[11] den Hollander AI, Koenekoop RK, Yzer S, Lopez I, Arends ML, Voesenek KE, Zonneveld MN, Strom TM, Meitinger T, Brunner HG, Hoyng CB, van den Born LI, Rohrschneider K, Cremers FP (2006). Mutations in the CEP290 (NPHP6) gene are a frequent cause of Leber congenital amaurosis.. Am J Hum Genet.

[12] Chenna R, Sugawara H, Koike T, Lopez R, Gibson TJ, Higgins DG, Thompson JD (2003). Multiple sequence alignment with the Clustal series of programs.. Nucleic Acids Res.

[13] Ramensky V, Bork P, Sunyaev S (2002). Human non-synonymous SNPs: server and survey.. Nucleic Acids Res.

[14] Ng PC, Henikoff S (2001). Predicting deleterious amino acid substitutions.. Genome Res.

[15] Ferrer-Costa C, Orozco M, de la Cruz X (2004). Sequence-based prediction of pathological mutations.. Proteins.

[16] Mátyás G, Arnold E, Carrel T, Baumgartner D, Boileau C, Berger W, Steinmann B (2006). Identification and in silico analyses of novel TGFBR1 and TGFBR2 mutations in Marfan syndrome-related disorders.. Hum Mutat.

[17] Ng PC, Henikoff S (2006). Predicting the effects of amino Acid substitutions on protein function.. Annu Rev Genomics Hum Genet.

[18] Schaeffeler E, Eichelbaum M, Reinisch W, Zanger UM, Schwab M (2006). Three novel thiopurine S-methyltransferase allelic variants (TPMT*20, *21, *22) - association with decreased enzyme function.. Hum Mutat.

[19] Gu SM, Thompson DA, Srikumari CR, Lorenz B, Finckh U, Nicoletti A, Murthy KR, Rathmann M, Kumaramanickavel G, Denton MJ, Gal A (1997). Mutations in RPE65 cause autosomal recessive childhood-onset severe retinal dystrophy.. Nat Genet.

[20] Thompson DA, Gyürüs P, Fleischer LL, Bingham EL, McHenry CL, Apfelstedt-Sylla E, Zrenner E, Lorenz B, Richards JE, Jacobson SG, Sieving PA, Gal A (2000). Genetics and phenotypes of RPE65 mutations in inherited retinal degeneration.. Invest Ophthalmol Vis Sci.

[21] Roepman R, Letteboer SJ, Arts HH, van Beersum SE, Lu X, Krieger E, Ferreira PA, Cremers FP (2005). Interaction of nephrocystin-4 and RPGRIP1 is disrupted by nephronophthisis or Leber congenital amaurosis-associated mutations.. Proc Natl Acad Sci USA.

[22] Henikoff S, Henikoff JG (1992). Amino acid substitution matrices from protein blocks.. Proc Natl Acad Sci USA.

[23] Perrault I, Rozet JM, Calvas P, Gerber S, Camuzat A, Dollfus H, Châtelin S, Souied E, Ghazi I, Leowski C, Bonnemaison M, Le Paslier D, Frézal J, Dufier JL, Pittler S, Munnich A, Kaplan J (1996). Retinal-specific guanylate cyclase gene mutations in Leber's congenital amaurosis.. Nat Genet.

[24] Dharmaraj SR, Silva ER, Pina AL, Li YY, Yang JM, Carter CR, Loyer MK, El-Hilali HK, Traboulsi EK, Sundin OK, Zhu DK, Koenekoop RK, Maumenee IH (2000). Mutational analysis and clinical correlation in Leber congenital amaurosis.. Ophthalmic Genet.

[25] Sohocki MM, Perrault I, Leroy BP, Payne AM, Dharmaraj S, Bhattacharya SS, Kaplan J, Maumenee IH, Koenekoop R, Meire FM, Birch DG, Heckenlively JR, Daiger SP (2000). Prevalence of AIPL1 mutations in inherited retinal degenerative disease.. Mol Genet Metab.

[26] Dryja TP, Adams SM, Grimsby JL, McGee TL, Hong DH, Li T, Andréasson S, Berson EL (2001). Null RPGRIP1 alleles in patients with Leber congenital amaurosis.. Am J Hum Genet.

[27] den Hollander AI, Davis J, van der Velde-Visser SD, Zonneveld MN, Pierrottet CO, Koenekoop RK, Kellner U, van den Born LI, Heckenlively JR, Hoyng CB, Handford PA, Roepman R, Cremers FP (2004). CRB1 mutation spectrum in inherited retinal dystrophies.. Hum Mutat.

[28] Janecke AR, Thompson DA, Utermann G, Becker C, Hübner CA, Schmid E, McHenry CL, Nair AR, Rüschendorf F, Heckenlively J, Wissinger B, Nürnberg P, Gal A (2004). Mutations in RDH12 encoding a photoreceptor cell retinol dehydrogenase cause childhood-onset severe retinal dystrophy.. Nat Genet.

[29] Banerjee P, Kleyn PW, Knowles JA, Lewis CA, Ross BM, Parano E, Kovats SG, Lee JJ, Penchaszadeh GK, Ott J, Jacobson SG, Gilliam TC (1998). TULP1 mutation in two extended Dominican kindreds with autosomal recessive retinitis pigmentosa.. Nat Genet.

[30] Hagstrom SA, North MA, Nishina PL, Berson EL, Dryja TP (1998). Recessive mutations in the gene encoding the tubby-like protein TULP1 in patients with retinitis pigmentosa.. Nat Genet.

